# Preliminary results of CO2 laser-assisted sclerectomy surgery (CLASS) in the treatment of advanced glaucoma in a Chinese population: Erratum

**DOI:** 10.1097/MD.0000000000008131

**Published:** 2017-09-15

In the article, “Preliminary results of CO2 laser-assisted sclerectomy surgery (CLASS) in the treatment of advanced glaucoma in a Chinese population”,^[1]^ which appeared in Volume 95, Issue 45 of *Medicine*, the authors would like to add the following acknowledgement, “We would like to acknowledge the S.K. Yee Medical Foundation for funding the purchase of the Trabectome machine and its surgical consumables.”
